# Rapid Ethical Assessment on Informed Consent Content and Procedure in Hintalo-Wajirat, Northern Ethiopia: A Qualitative Study

**DOI:** 10.1371/journal.pone.0157056

**Published:** 2016-06-03

**Authors:** Serebe Abay, Adamu Addissie, Gail Davey, Bobbie Farsides, Thomas Addissie

**Affiliations:** 1 Ethiopian Public Health Institution, Addis Ababa, Ethiopia; 2 Brighton and Sussex Medical School, Brighton, United Kingdom; 3 Addis Ababa University, Addis Ababa, Ethiopia; University of New South Wales, AUSTRALIA

## Abstract

**Background:**

Informed consent is a key component of bio-medical research involving human participants. However, obtaining informed consent is challenging in low literacy and resource limited settings. Rapid Ethical Assessment (REA) can be used to contextualize and simplify consent information within a given study community. The current study aimed to explore the effects of social, cultural, and religious factors during informed consent process on a proposed HPV-serotype prevalence study.

**Methodology:**

A qualitative community-based REA was conducted in Adigudom and Mynebri *Kebeles*, Northern Ethiopia, from July to August 2013. Data were collected by a multi-disciplinary team using open ended questions concerning informed consent components in relation to the parent study. The team conducted one-to-one In-Depth Interviews (IDI) and Focus Group Discussions (FGDs) with key informants and community members to collect data based on the themes of the study. Tape recorded data were transcribed in Tigrigna and then translated into English. Data were categorized and thematically analyzed using open coding and content analysis based on pre-defined themes.

**Results:**

The REA study revealed a number of socio-cultural issues relevant to the proposed study. Low community awareness about health research, participant rights and cervical cancer were documented. Giving a vaginal sample for testing was considered to be highly embarrassing, whereas giving a blood sample made participants worry that they might be given a result without the possibility of treatment. Verbal consent was preferred to written consent for the proposed study.

**Conclusion:**

This rapid ethical assessment disclosed important socio-cultural issues which might act as barriers to informed decision making. The findings were important for contextual modification of the Information Sheet, and to guide the best consent process for the proposed study. Both are likely to have enabled participants to understand the informed consent better and consequently to comply with the study.

## Introduction

Prospective medical research participants may have experienced a range of traditions of healthcare and hold various beliefs about illness and causation of illness. Participants will often be unfamiliar with the concept of research and may be sensitive to some practices, such as taking blood samples. Local, social, cultural and economic contexts are therefore critically important when research is designed [1,2].

International research collaborations now commonly include investigators and participants from developing countries. While international ethical guidelines may govern these collaborations, in reality, these take very little account of the different social structures, cultural norms, legal frameworks and methods of communication to individual study participants or the wider community [3].

In Ethiopia, research is governed mainly by principles and regulations established by regulatory authorities such as the Ethiopian Science and Technology Commission (ESTC), now Ministry. The problem is not lack of regulation, rather the generic nature of international guidance on informed consent form content. This has given rise to criticism of the western approach to identifying and addressing ethical issues related to research in developing country settings [4]. The inflexible use of international ethical guidelines in health research has prompted investigators to seek an approach to adapting these guidelines to local situations [5,6].

Information gathered from patients, researchers, fieldworkers and potential participants can help to shape the research agenda as well as the consent process for subsequent studies. This information may suggest how to explain a research project, how decisions to participate may be reached, and how true respect for participants can be fostered [2]. Various studies conducted in developing countries have emphasized the importance of addressing the local context while respecting absolutely the universal ethical standards[7], especially in the developing world where there is a difference between what is assumed by researchers and the practical reality on the ground [2,8,9]. A major challenge for researchers is to deliver complex information at the right time in appropriate language and style. To this effect, informed consent documents should be adapted to the local culture and the educational level of the population [10]. Bull and Farsides recognized that problems often arose when ethics committees in low-income countries made decisions based on international guidance that either missed issues of importance locally or over-emphasized issues that had little ethical importance outside a western perspective [9].

A qualitative study done in Ghana used a rapid assessment tool designed to inform the adaptation of interventions to local cultures and conditions. Result of the study showed that MalariaGEN participants and field staff seeking consent were generally satisfied with their understanding of the project and were familiar with aspects of the study relating to malaria [8]. This tool has become known as Rapid Ethical Appraisal or Assessment (REA—a brief qualitative intervention designed to map the ethical terrain of a research setting).

More recently, the Rapid Ethical Assessment approach was used prior to genetic studies in Southern Ethiopia and North West Cameroon, to inform consent form development and the processes of seeking consent from the community and individual participants. Approaching study subjects via locally trusted individuals and preceding individual consent with community sensitization were considered the optimal means of communication [2,11,12].

The current study aimed to explore the effects of social, cultural, and religious factors in the informed consent process of a HPV-serotype prevalence study. The parent study planned to use vaginal swabs and blood collection [13], which together with possible gender and culture related barriers suggested that Rapid Ethical Assessment would be essential before the actual study.

## Ethical considerations

Ethical clearance was first obtained from the Research Ethics Committee (REC) of Addis Ababa University School of Public Health(AAUSPH). Following endorsement by the REC, the Tigray Regional Health Bureau (RHB) was informed about the objectives of the study through a support letter from AAUSPH. After reviewing the proposal, the Tigray RHB wrote a permission and support letter to Hintalo-wajirat district. Then Key Informants were asked for verbal consent to confirm informed voluntary participation. Both the current study and the proposed parent study were approved by Research Ethics Committee (REC) of AAU, who considered that since the current study was non-invasive and used only interviewing of participants, verbal consent would be adequate. An independent community member acted as witness for voluntary informed decision making of participants to take part in the study.

## Methods and Materials

### Study setting

This study was conducted in Hintalo-Wajirat district, South Eastern Tigray, Ethiopia from July to August, 2013. This district is adjacent to Mekelle, the capital city of Tigray, and a favored research center for Mekelle University and other colleges within the city. The total area of the district is approximately 1,393 square kilometers [14]. The district comprises of 22 *kebeles*(The smallest administrative region in Ethiopia). According to the Central Statistics Agency (CSA) report of 2007/8, the population is estimated to be 152,219. Of this, males constitute about 75,262 (49.4%) and 140,291 (92.2%) of the total population live in the rural areas [14].

### Study design

A qualitative community-based rapid ethinographic assessment was conducted to explore biomedical research and informed consent on the context of the study community. Two *kebeles* were purposively selected where the parent study was planned to be conducted. We used open-ended guide questions developed based on socio-cultural determinants and components of biomedical research ethics in relation the parent study (Annex I). The parent study was entitled “HPV-Subtype Prevalence Study" and planned to use blood and vaginal swab sample collection, and reproductive health interviewing among healthy pregnant women during their antenatal care visit at health facilities. Since invasive and sensitive procedures were planned, it seemed important to conduct rapid ethnographic assessment before the actual study.

Participants were selected using purposive and convenience sampling methods to include different segments of the community. We included religious and community leaders, field workers, Rural Health Extension Workers (RHEWs) and other residents of the study area.

## Data collection and analysis

Data were collected by a multi-disciplinary team composed of one MSc in social anthropology and two MPH graduates. The local leaders suggested key informants and guided the team on socio-cultural events in the community. Three of the team members were native speakers of Tigrigna, the local language, and two of them were familiar with the study area. The data collectors had substantial qualitative experience and were given orientation about the interview and the parent study. Study team introduced themselves and objective of the study before each interviews and group discussions. All data collectors were male except one female community health workers leader who was involved to guide the team but no role in data collection and analysis.

We conducted the Rapid Ethical Assessment based on eight thematic areas: knowledge about biomedical research and treatment, terminologies of cervical cancer, the consent procedure, sample collection, communication channels, gender dynamics of decision making to participate in research, participant selection, and their expectations from taking part in the study. These thematic areas were developed *priori* to data collection and analysis. Out of the 45 participants included in this study: three were administrative leaders, three were religious leaders (two Orthodox and one Muslim), six RHEWs, two field supervisors, one women’s association leader and the rest 30 were non-leader residents of Hintalo-wajirat district. We assumed that minority segment of the community somehow represented by the non-leader residents who were interviewed while they were visiting health facilities, attending community meetings, participating in community development activities, and collecting fertilizer from agricultural offices.

We did a total of 11 recorded IDI (at least one from each segment of the community mentioned above) and 5 FGDs(includes two women groups, one men group, one mixed group, and one group of RHEWs and field supervisors). Each group included 6–8 participants of different age group. We used both homogeneous and heterogeneous groups for the FGDs to enhanced credibility through free discussion among the participants and to check any gender based differences. We looked at consistencies of concepts between the one to one interviews and group discussions, and among the different FGDs. Transferability were also checked by triangulation of interviews with observation data. A tape recorder was used for recording the IDI and FGDs. At the end of each day, the team debriefed the assessment, took corrective measures where necessary and planned for the next day. Team members also participated in a range of social activities, including markets and local meetings, to collect socio-cultural data by observation. After sufficient data had been collected, they were transcribed in Tigrigna and then translated into English.

Data analysis was done as a separate stage following data collection and used open coding software[15–17]. Although the study period is short we used a kind of explorative ethnographic study focusing on subjective experience and interpretation of research and treatment in relation to parent study in the particular setting and context. First the PI read the complete transcripts and generated a list of codes. Then after aggregating and defining concept, the PI developed memos to elaborate the concepts or categories developed. The findings from observation and notes from debriefing were also included in the analysis. Finally, integration of categories was done by linking them to the themes by using techniques including rereading memos, immersion in the data, creating descriptive sentences and drawing diagrams.

## Results

### Socio-demographic characteristics of participants

A total of 45 people participated in the IDIs and FGDs of whom 31 were female. Each group discussion included 6–8 participants, and lasted an average of one hour, while IDIs took on average 30 minutes. The age of the participants ranged from 19–62 years. The participants included: three administrative leaders, three religious leaders, two health workers at health facilities, six RHEWs, two supervisors, one Women’s association leader and residents of Hintalo-wajirat district. Most of the participants(19/45) had no formal education,16/45had 1–12 years’ schooling and 10/45 had a diploma or further degree.

### Understanding the difference between health research and treatment

After introducing the purpose of the proposed parent study and assuring confidentiality, each interview was started by asking about their understanding of health research and treatment. We asked participants if they could differentiate research from treatment. Many of the participants found it difficult to explain what biomedical research was. Differences were noted between the rural residents(farmers, merchants, housewives)and administrative and community leaders: rural residents and religious leaders tended to have experienced research in the context of agricultural studies and thought of biomedical research as any laboratory investigation to diagnose disease and guide patient treatment. The administrative leaders, community health workers and field supervisors were better able to differentiate research from treatment. A female rural resident of the study area said:

“*…research is like to identify the type of water and soil*, *for what purpose the soil is important*, *for what purpose the land is important*, *what type of fertilizer would be appropriate*, *what type of medicine is needed*? *In this way I understand what research means*.*”*

(Female, 45 years, house wife, IDI)

A 52 year old priest and religious leader during the IDI stated:

*“Ehh…Research is ehh*. *Yes… it is about…*[difficulty of explaining], *it is about medical follow up*. *It is not to miss once using medicine*. *Research is about study; what is the strength…to know how the past time was look like and what is coming*.*”*

*“Research is an investigation of a disease*, *for example if a mother comes to a health center*, *first investigation should be done on the blood*, *urine or stool*. *If the disease is known*, *then medication will be given to her*.”

(Male, 50 years old, farmer, IDI)

*“Research is investigating what disease occurred*, *what the disease is*, *and what causes that*. *You should not get treatment before investigation*. *Medicine can treat you only after the disease has been studied*.*”*

(Male, 60 years old, board retired, FGD)

*When somebody come to health facility for investigation and get treated*, *we say treatment*. *But research is when you go to the community to investigate a problem by taking a sample of participants*. *Whereas*, *when a mother comes directly to health facility and gets medicine*, *we can say treatment*.

(Female, age 28 years, community health worker, IDI)

### Awareness of and local terminologies for cervical cancer

Almost all participants had heard of cancer and some thought that a wound could change into cancer unless treated early. Participants in the IDIs and FGDs mentioned that they had got information about cancer from TV, health professionals and rural heath extension workers. Although a range of terminologies for cancer were known by almost all of the participants, the knowledge and perception given to cancer varied among participants. Female participants thought that a wound could change to cancer unless treated quickly. Male participants believed that cancer was any previously existing swelling that had been treated with herbal ointment.

The research team observed that there were different community-based teams which included the1 to 5 and 1 to 25 health developmental armies, and Gujile-Limat (development teams with community leaders who were accountable for the community health workers). The community health workers were also accountable to the nearest health center. These teams mainly discussed maternal and child health issues. In addition, leaders of these community based teams were responsible for reporting any new case of disease, including breast lumps, and referring the patient to health facilities. Cervical cancer was little known by the community in the study area. Although some female participants were aware of cervical cancer, they explained that it was difficult to see a doctor due to embarrassment. Overall, participants of FGDs of both sexes agreed that cancer is a non-curable and serious disease, contrasting it negatively even with HIV. A 50 year old male IDI participant, who was resident in the area for 10 years said:

“*It existed previously*. *It is called ‘****Menkersa****’*[any persistent swelling over the body with blood and pus-like discharge]. *One time on market day*, *Saturday*, *here in Adigudom*, *Dr*. *XXX educated us about cancer and TB going around by a car*, *and said ‘cancer is a worse disease even than HIV*, *and has no treatment*.*’ I heard this and understood its seriousness*. *So the communities understand that cancer is a dangerous disease and there is no cure for it*.*”*

A male FGD participant explained:

“*Now*, *the term ‘****Menkersa****’ is known*. *But previously*, *for example when mothers developed breast swelling*, *they called it something else*. *They used to treat it with herbal medicine*. *Then the disease became disseminated and worsened*. *Now the disease causes much harm*. *If a mother has breast cancer*, *it cannot be cured*. *It was previously called* ***boils***, ***swelling*** *(malignant) or* ***kintarot***[a general term given to swellings, mostly to anal warts or hemorrhoids].

“*Now*, *about cancer*, *we listen to the radio and watch cancer on TV; we see that they get cancer of the breast*, *we see how it inflames the breast*, *and we see how it disseminates*. *But we don’t know about cervical cancer*.”

(Male, 62 years old, driver, male FGD)

*“Most of the girls do not explain like in other parts* [of the body]. *If it is in other parts* [outside the uterus], *they can explain it well*, *it can be easily examined*. *Since it is inside of the uterus ehhi… it is just shame*, *shame to tell for others*. *I knew two-three women harmed by the disease in the uterus due late to treat*. *But now the concern is increasing*, *it means that if you get a wound*, *it will change into cancer*.*”*

(Female, 40 years old, house wife, female FGD)

### Information dissemination and communication channel

The most accepted information communication channel was through religious leaders. Health professionals and community leaders like *Bayto*(Selected community leaders offering justice services) were also good channels for dissemination of information to the community. Political administrative leaders should be involved only for legal concerns. Going directly to the target individuals might decrease acceptance rates, since participants might relate the study to political or religious agendas. A female participant aged 40 years said:

“*Bayto are preferable*, *but for health studies*, *health professionals are better*. *Others*, *like community leaders also have the chance to meet the community*.”

*“It is eh… here there are three types*: *the administrators*, *religious leaders and health professionals should discus together how the information should be disseminated to the community*.*”*

(Male, 45 years, priest)

“*Religious leaders are the most accepted*, *more than community leaders or administrative members of the kebele*.”

(Female, 37 years, house wife)

Participants in the male FGD mentioned distrust of newcomers who sought community involvement and might have hidden agendas.

*“I think* [the approach should be] *through community representatives or health professionals*. *The people always hesitate with those with new hair* [newcomers]. *For example last year there was an organization which helped with eye glasses*, *but later on*, *we found out that they had a hidden religious mission*. *So the community always fears that the newcomers could have hidden religious or political agendas*. *Even we have been told in a meeting to be careful for our peace and to report immediately any suspected religious extremists*.*”*

(Male, 52 years, board retired, FGD)

*“Yes I agree with what my friend said*! *Now we are giving you information because you come with Sr*. *XXX* [health extension worker of the district]. *She is our representative and we believe that she couldn’t bring us an illegal person*. *Of course it is our fault*, *we have to ask you who you are*(letter of permission). *But we see her with you and you look like us—you speak Tigrigna*. *That’s why we simply start our discussion*.”

(Male, 30 years, construction worker, FGD)

### Consent seeking procedure

We asked respondents the best way to seek participant consent, for example using a signed written document or through verbal agreement. Most participants explained that signing was not a good approach because potential participants might relate this to legal accountability. Signing might also prevent participants from explaining their true feelings. The research team also observed signing of individuals while attending meeting, taking food ration for their children supplied by world food programme, and during fertilizer and seed distributing among farmers. Residents of a village may be obliged to participate in water and soil conservation, reforestation or fertilizer utilization. In such activities, it is common to request the signature of participants to follow and trace whether or not they have discharged their duties and responsibilities properly. Signing an attendance sheet is used as evidence that participants were informed of the campaign activities and may be used to account for any failure of planned activities. In addition, there are meetings for performance and attitude change evaluations. Due to these and related concerns participants are afraid of signing regardless of the purpose and the situation. They do not trust the investigator even if assured that signing to participate is confidential. If they sign, they will be careful about the information they give, and are more likely to reflect on positive aspects of the issue to be discussed. FGD participants also thought that using verbal consent helped participants to respond more freely during the interviews.

“*We don’t suggest using signature—they are used for other purposes*. *Asking someone to come and sign may hinder their involvement*, *and may make it difficult to get genuine information even from those who have voluntarily signed”*.

(40 years, Female, women’s association leader, IDI)

“*Signing could be difficult*, *as community members worry about what may happen to them after signing*. *She may think that what will happen to her after signing*? *Since it is for health*, *they can participate freely*. *If we explain to them verbally that we are going to investigate for cancer*, *no one will be afraid or refuse*. *Therefore*, *verbal consent is preferred to written”*.

(Female, 28 years old, urban health extension worker)

On the other hand, some participants assumed that taking part in a research project was an individual’s obligation and that participants should sign in order to accomplish the research project successfully. A 45 year old priest in the IDI explained:

*“Yes*, *it’s better to sign*. *For most of the community*, *signing is better*. *Let’s say*, *you have said ok to perform some activities*. *Let say you appoint me to meet you at some fixed time but maybe I don’t attend on time*, *then there will no means to punish me*. *But if I have signed*, *I can’t withdraw and you will be successful*. *But to do this*, *first you have to conduct education*, *counseling and meetings with the community*.*”*

### Sample collection technique

We asked about potential concerns around giving blood and vaginal secretion samples. Most participants said that they would prefer female health professionals to conduct reproductive health interviews and take vaginal samples. Since the participants would be healthy and not in labor, they might feel embarrassed with a male data collector. But some male participants in the IDI explained that as long as the data collectors were health professionals and the study was at a health facility, the gender of the data collectors would not be a big deal. While there was no data collector gender preference for blood sample collection, some people thought that giving blood might be associated with an HIV test or fear that the result might show cancer. The female FGD participants explained that a female sample collector would be preferred to a male.

*“A female for female is like herself*. *It is better to be same gender*. *It is easy for a woman to explain all her secrets to a woman but difficult to explain to a man*.*”*

(Female, 45 years old, housewife, FGD)

*“For the female* [the data collector] *should be female and for the male should be male*. *The female would be able to explain all her problems only if it is to a woman*.*”*

(Female, 29 years, merchant, FGD)

Participants in the male FGD also support the preference for female data collectors.

“*I…yes*, *to participate confidentially; I think female sample collectors would be preferred*. *If she is a woman like herself*, *she will not be shy; it is for her health*. *If it is a male sample collector*, *she may feel ashamed*.”

(Male, 62 years, farmer, FGD)

“*Yes*! *eh*, *the main thing is they have close contact with females*. *Health extension workers*, *who are females*, *are easier to talk than someone like me*. *If someone is in a difficult situation or they are sick and if it’s their last option*, *it doesn’t matter who sees them*, *but in a healthy condition they’re more likely to confide in a female*.”

(46 years old, male, community leader, FGD)

For some participants, it was difficult to communicate what vaginal secretion means. Some thought it meant menstrual discharge and others as fluid secreted during orgasm. Using the phrase *‘the natural vaginal wetness or fluid’* made communication with study participants easier. The term ‘HPV’ had no local equivalent and was confused with the acronym ‘HIV’.

“*Is vaginal secretion to mean menstrual discharge*? *How it could be taken*? *No problem to give blood sample since there is testing for HIV every year*. *Even we religious leaders have taught the community to be tested for HIV*.”

(Male, 47 years, priest, IDI)

*“The vaginal secretion could be difficult*, *how could they give a secretion without their husbands*? *Haha… (Laugh)”*

(Male, 52 years old, merchant, FGD)

### Gender dynamics surrounding decision making

Most respondents were clear that the decision of a woman to participate in the study was hers alone—she could make decisions about any aspect of her life. However, some mentioned circumstances in which it would be beneficial that the woman discussed with her husband, for example HIV testing or choosing a family planning method. Some participants recommended the husband’s involvement in decision making so that the idea could be shared and the partner involved in any future tasks to be performed by the woman. A 28 year old female community worker explained that:

*“The decision to participate is made mainly by the woman herself*, *but her husband also has a role in decision making*, *for example when they come for HIV testing*. *In this study*, *it is better to discuss with their partner even though the woman could decide alone*.*”*

(Female, 28 years, IDI)

Participants from the male group FGD replied:

*“The decision maker is the woman herself*. *For example*, *to participate in this discussion today*, *I didn’t ask my wife’s permission*. *Likewise the woman can decide whether or not to participate in any study*. *But being a supportive partner*, *to share ideas and to know my opinion*, *my involvement might be important*. *Otherwise the decision-maker is the woman herself*.*”*

Participants in the female FGD explained:

*“She is the decision maker*, *which means me in myself*, *no one should decide on behalf of her*. *She should have self- confidence*.*”*

(Female, 30 years old, Housewife)

*“That is the fact*, *pregnant women are the owners of their health*, *and nobody should interfere with them*. *If it is for other investigations*, *she may need to convince her husband but in this case it is for her own health*.*”*

(Female, 25 years old, Merchant)

### Participant selection

Selecting participants without explaining the selection system used might lead to negative consequences among both included participants and excluded community members. Those included may worry that they were chosen based on their health condition and suspect that they have the disease being studied. Those excluded may feel that they have missed some benefits of participation in the study or other safety net programs (like that of the World Food Program). So it was vital to create awareness about the research, its purpose and the participant selection method through health extension workers, religious and community leaders. A 40 year old, female resident of Adigdom town, during the IDI responded as:

*“Yes*, *I may feel or think something if I am not asked*. *However*, *it is very difficult for you to include all the people*, *as you need only some*. *Here you have the health workers and community leaders*. *They always have monthly meetings*. *So you need to convince the people through them*.

*Let’s say two pregnant women went to a health facility together*. *If one is examined in the study and the other not*, *what would happen is*, *the one included in the study will think*: *what a different thing has happened to me*? *What they have seen on me*? *What they heard about me*? *We came together but only I was invited to be investigated*. *The woman excluded also thinks she has been treated differently and unfairly based on something*. *Both could have negative feelings*.*”*

(Female, 45 years old, housewife, FGD)

Participants also asked what to do to avoid such challenges during participant selection. They agreed that using the lottery method in the presence of potential participants was the best method of participant selection and the community used this method in different social activities.

*“One thing*, *there are women community teams (Gujile-limat) with one leader*. *So invite the team leader and orient them on how the process of selection should performed*. *They can easily make things clear and convince their members*.*”*

(Male, 42 years, farmer)

*“It is better to use the lottery method in the presence of all the members of the team*. *Otherwise it may have a negative impact on the community*. *It may even be bad for future relationships between the team leaders and members*, *who may consider the leaders to have acted unfairly*.*”*

(Female, 45 years old, housewife, FGD)

### Expectations from participation

In health-related research, participants do not expect any immediate benefit; instead they worry about their test results and the need for treatment according to their test results. However, there are elected community leaders engaged in community mobilization and sensitization that experienced with payment for their involvement.

*“In health related studies the community has no incentive expectation*. *But for example in household surveys*, *they may have expectations related to the ‘safety net’ and different aid programs*. *In health*, *they have no expectations relating to benefits or incentives*. *So many studies have been done*, *but until now I don’t see any feedback from the investigators*.*”*

(Male, 30 years, district administrator, IDI)

*“Yes*, *of course incentive needs are still present*. *In the recent study on immunization by the World Food Programme*, *those who mobilized the community were paid about 70 birr* [equivalent to 3.5 USD] *per day*. *So if incentives are given*, *nobody resists and there is an assumption of benefits from participation*.*”*

(Female,28 year old, Health Extension Worker, IDI)

## Discussion

The rapid ethical assessment described here revealed a number of issues relevant to the ‘parent’ study, a survey of HPV- serotype prevalence. Problems regarding informed consent are usually created when the researchers and the potential participants are from different cultural and religious backgrounds [18]. Identifying and addressing such issues is part of the investigators’ responsibility. Researchers should pay great attention to these issues so that participants are well informed and have good comprehension of the informed consent documents that they are required to sign. Furthermore, researchers should take all the steps necessary to ensure that participants fully understand what is being stated in the consent form [19].

The rapid assessment enabled us to modify the information sheet and consent procedure to the local culture and the educational level of the study population. Other researchers have employed the same approach to optimize consent information for research in low income and resource limited settings[2,8,9,11,12].

One of the difficulties that investigators and other research personnel often encounter during informed consent process is “therapeutic misconception” which can render informed consent invalid [20]. The therapeutic misconception occurs when a research subject fails to grasp the distinction between clinical research and ordinary treatment and attributes therapeutic intent to research procedures [21]. The therapeutic misconception matters ethically because it is incompatible with truly informed consent and thus undermines the research enterprise. Disregarding, fostering, or taking advantage of misplaced trust may affect subjects’ self-trust and their perception of researchers, disturb other trust relationships, and erode public confidence in biomedical research [22]. In our study, therapeutic misconception was a big deal in rural resident participants, farmers, house wives and religious leaders. Most of the participants understand health research in terms of laboratory investigation to diagnose a disease, while others has explained research relating to land and agricultural farming, which coincides with other study findings[11,12,23]. This could be due to the fact that most of the people in Ethiopia are engaged in agriculture and have experience related to agricultural research and best practice expansion among farmers. In the other hand, administrative leaders, community health workers and supervisors had better understanding of research and treatment. This could be due to their previous exposure to a research as a participant or as a facilitator of community based research. These findings were in line with an interventional study conducted in Malawi; information was given in the context of the language, culture and beliefs of the society where the proposed research would be conducted [24,25]. Explaining health research in terms of agricultural studies, for example sowing in lines and using fertilizer to make farmland more productive, may be helpful for participants to understand what health research mean in the rural parts of Ethiopia.

Research in developing counties is influenced by many barriers to ensure participant understanding. Language barriers often present difficulties during informed consent in developing countries. Problems can arise not only during the process of obtaining consent from an individual participant, but also in the process of translating the documents. Misunderstandings can occur because of incorrect or inadequate language translations [26,27]. Efforts to improve the quality of informed consent, like simplifying and adapting consent protocols are necessary, especially when dealing with cultures and languages in communities with previously unexposed to biomedical concepts and terminology [28]. There is no logical reason to insist that informed consent be identical in countries with markedly different cultures, socio-traditions, and literacy [29]. Ethiopia is a country having more than eighty ethnic groups with different language, culture and traditional believes in which ethical appraisal and socio-culture mapping might be important for research and health care practice.

In our study we found several local terminologies used for cancer. *‘Menkersa’* and *‘kintarot’* were most frequently mentioned. These terms were used for any externally visible malignancy on the body for which herbal ointments are used. The name *‘Menkersa’* is also related to something bad which cannot be avoided easily. Breast cancer was the type of cancer that most participants were familiar with, through television programmes, but they had little awareness about cervical cancer. There were also misconceptions surrounding the genesis of cancer, including beliefs that any wound changes to cancer unless treated rapidly. Other participants trusted in herbal ointments to treat any surface malignancy, which might have a negative impact on early management of cancer. Other studies have shown that misconceptions related to the etiology of cancers have a negative impact on health seeking behavior in Ethiopia [30,31].

A bond of trust should develop between researchers and the community as complementary partners in the research enterprise to improve informed comprehension of research participant and ideally lead to increased recruitment and retention rates [4]. Respect for cultural traditions builds such a foundation of trust between researchers, study participants and the local community. Researchers should identify concerns that are culturally tailored and develop strategies to address them in a meaningful way. If possible, investigators should consider developing alternative methods for achieving successful results[32,33].

The most accepted way of information dissemination to and communication with the communities were through religious leaders. Health professionals and community leaders (*Bayto*,*Tena-fana*) were also good channels for information dissemination to the community. The researcher going alone to the community may experience difficulty in communication and lose the trust of the community, who may be suspicious of hidden agendas behind the research objectives. On the other hand, over-reliance on religious leaders and health professionals might result in undue influence to participate in the study without genuine understanding. In our case, participants explained that they did not need to ask who we were, if we approached them with local health professionals.

Obtaining valid and appropriate informed consent is both an ethical and legal requirement to protect the rights and welfare of human subjects. The method by which consent is confirmed (written or verbal) is left open by many ethical review committees. Participants generally advised not insisting on signatures as this might act as a restraint on what participants would divulge, and also stated that signing the consent form was less important than ensuring that they understood what was involved in the research.

Verbal consent may be acceptable for research participants who are illiterate and, therefore, cannot read or sign informed consent forms. In some regions, participants may be unwilling to sign consent forms in the belief that they are signing away rights, or that other adverse repercussions may follow. If requesting that participants to sign consent forms is inappropriate, other means of recording their consent to participation is required; for example, audio taping or use of an independent witness to observe the verbal consent. Sometimes the research worker who is informing the participant will sign a form stating that the appropriate information was given and verbal consent received [3,34,35]. Tekola, et al. found that the use of written consent forms in less literate populations may unfairly exclude potential participants who cannot read or write, and may create confusion and anxiety particularly for potential participants who are unaccustomed to signing documents [2]. Performing Rapid Ethical Assessment may be helpful in order to identify the optimal approach for a given community, which can then be approved by the respective ethical review committee.

The parent, HPV-serotype prevalence study, required collection of blood and vaginal secretion samples. In the REA participants explained that prospective parent study participants would be more comfortable if samples, particularly the vaginal secretion samples, were collected by female health professionals. There was no particular gender preference for blood sample collection; however, some had reservations about giving blood for the HPV test as they associated this with past experience of giving blood samples for HIV testing. We did not find a precise local term for Human Papilloma Virus (HPV), and the acronym ‘HPV’ was confused with ‘HIV’, so it was unsurprising that the test for HIV came to mind when people were asked for a sample for HPV testing. Participants were also afraid that their HPV test result might show cancer which could not be treated.

The results of the rapid ethical assessment were used to shape the information sheet used for the parent study, in particular to minimize confusion between HIV and HPV. Data collectors were trained to explain the difference and then ask potential participants to explain this back to them. They used phrases like “…I am not talking about HIV, unlike for HIV, you will not get back your HPV test result. The benefit of this HPV sero-prevalence study is to produce a vaccine against HPV so that the next generation of girls can be protected from cervical cancer…”

In low-literacy settings, potential study participants can be helped to understand key components of consent information using innovative tailored local narratives [24]. In our case, measles is a well-known disease in Tigray, and in the study district. Most of the communities were also familiar with agriculture research to test a new seed, a new fertilizer, and a new method of ploughing. Based on such findings of the rapid ethical assessment, the team developed a narrative explanation to help potential participants in the parent study easily understand the consent form (Annex II).

Obtaining genuine informed consent from research participants is best thought of as a process of sharing information and addressing questions and concerns, rather than simply obtaining a signature on a prescribed form. It starts with the researcher developing an awareness of national or regional guidelines, and may involve discussions with the community and/or family members of potential participants. Participants must then give their individual consent to participate on an informed consent form developed specifically for the research project. Obtaining the agreement of local community leadership for the proposed research is almost always good research practice and is mandatory in some communities [36].

In this rapid ethnographic assessment study, we found that prospective participant(pregnant women) of the parent study could decide alone. Even though the decision is the right of the participant, there were a lot of circumstances that the male partner should be involved; for example, during choosing family planning method, testing for HIV and other investigations. Many participants agreed on their partner involvement or asking their opinion would be help full during female recruitment for the study. This is an important finding of this study in contrary to the current notion which gives emphasis on individual autonomy for decision making for participation. Studies showed that women were more than likely to reporting obtaining permission from someone else before participating than men do [37, 38]. Biomedical research participants from developing countries need to discuss with their spouse, families and friends before decision made for participation. This can be done by giving time for discussion with or through invitation of the person they assumed that should be involved in the informed consent process. Therefore, it is worthy to consider partner or family involvement during informed consent decision making process based on the socio-cultural and gender dynamics on decision making of the study setting.

Other factors needing emphasis during participant recruitment are the participant selection process and participant expectations from the study. Groups or communities invited to participate in research should be selected in such a way that the burdens and benefits of the research will be equitably distributed. The exclusion of groups or communities that might benefit from study participation must be justified. Subjects should be drawn from the qualifying population in the general geographic area of the study without regard to race, ethnicity, economic status or gender unless there is a sound scientific reason to do otherwise [7]. In our study we found that participant selection could result a negative consequences among both those included participants and excluded community members. Those included one may think that they were selected based on their health condition and suspect that they have the disease to be studied. The excluded community members may also feel they are being unfairly treated if they are unable to receive the benefits from participation. This may disturb the researcher and community relationship in future research activities. These community misperceptions about participant selection may be handled through awareness creation about the research, its purpose and participant selection method by using health extension workers, religious and community leaders.

Participants of biomedical research could have different expectations of their involvement in research. Some may simply want to help the researcher by giving information that they think the researcher is looking for, while others may expect some benefits from their participation. In the current study setting, participants didn’t expect any immediate benefits like incentives or money for their participation. Instead, they were more worried about their test results and the need for treatment according to the result. Some interviewees complained that studies done previously had not given feedback as the researchers promised.

The Rapid Ethical Assessment enabled the parent study to use the most important findings to modify the information sheet, consent form and consent process. Based on the study findings, the research team suggested alternative local terminologies that could be used for cancer, explained ways of minimizing confusion between HPV with HIV, justified taking verbal consent over written consent, recommended that data collectors were female, and suggested a way of explaining the main aim of the study. Other researchers have used Rapid Ethical Assessment to inform the consent process for parent studies in similar ways [2,3,8,11,12].

Although efforts were made to ensure credibility, dependability and transferability of the study by using qualified reach team, different participant groups and good methodological triangulation, this study has potential limitations. In this study we used only male data collectors, which may have made some female participants less likely to discuss gender-sensitive issues. The study covers a limited geographical setting over a short period of time, but this was deliberate given the specific needs of the parent study. Other ‘parent’ studies may require Rapid Ethical Assessment in other geographic and cultural settings to explore the factors affecting informed decision making relevant to these studies.

## Conclusion

The Rapid Ethical Assessment in this study revealed a number of issues concerning knowledge and perception of research, gender dynamics, decision-making, and information communication channels. Understanding the socio-cultural dynamics of a study setting, approaching participants through trusted community members and using local narration techniques can all optimize informed decision making.

## Annex I

Interview guide questionnaire for IDI and FGD used for Rapid Ethical Assessment.

***Introduce yourself and about the study:***—Hello /Greetings! my name is __________. I ama researcher and member of a team from Addis Ababa University School of Public Health. There is a proposed study to know more about cancer diseases in Ethiopian women. Now we are collecting a base line assessment before conducting the actual study. So we are going to discuss with you about the proposed study regarding to this society. The information you give us will be recorded to put on words later. All the information you give us and the records will be kept confidentially.

1. Would you please tell us your age, job, educational level and duration for how long you live here? **No need to mention your name.**
2. What do you know about research? How you understand medical research? What about medicine or treatment mean?
3. What do you know about cancer? What about cervical cancer?
4. Would you please tell us the local name given to cancer if any!
5. As I explained you earlier there is a proposed study to find out more about cancer disease in Ethiopian women. Cervical cancer is the most prevalent and mostly associated with human papilloma virus and other co-infections of the genital or reproductive organ. For this study pregnant women who have antenatal follow up in health facilities will be interviewed, and small drop blood and vaginal fluid will be collected. The findings of the study will help the government to introduced vaccine against cervical cancer in the country. Therefore, future generation of girls will be benefit. How could we get volunteer pregnant women participant in this study?
6. Which type of consenting method do you think preferable? Verbal or signed consent? Why?
7. Who decide for participation in a research in the family members? Who decide the pregnant women to participate in this proposed study? In general what is the decision making power of women in this society?
8. What could be the possible barriers for pregnant women to give a small amount blood and vaginal secretion? Cultural or religious effect if any.
9. How can we help pregnant women to understand consent and participate voluntary? Who do you thing better to collect the samples? Male or female health professional?
10. During participants selection what could be the effect of including some participant in the study and others not? What about the participant expectation in response to participation in a research?

## Annex II

Narrative explanation to be annexed with the information sheet in the parent study.

**Research:** is a work trying to find new facts. For example a farmer wants to use new method of farming like sowing in line or to use fertilizer to make his farm land more productive. So first he tries the new method or the fertilizer in a plot of land. If the product is better enough then he use this method for the whole of his farm land. This best experience will be expanded to his neighbors.

**Medical research:** In a medical research is also the similar. If the findings from few study subjects are important for health, then these will be used for the general public. It is different from treating of the individual patient. As you know before many years there was no vaccine for measles (Nifyo, local language). Many children got blind and died due to this disease. Now, after research did vaccine for measles has found and many children saved from blindness and death by vaccination. Similarly this study is to introduce vaccine against “Human Papilloma Virus” and to prevent cervical cancer. Then future generation girls could be saved forms this incurable disease. Though you are not directly benefited from involving in this study, you are paying remarkable contribution for your daughters and next generations.

**Used local words for cancer: “Menkersa”** (very common), **cancer** (sometimes) **“Kintarot”** (rarely)
